# Supplementary material for: Evolutionary origin of genomic structural variations in domestic yaks
Source: Nat Commun. 2023 Sep 19;14:5617. doi: 10.1038/s41467-023-41220-x (PMC10509194; doi:10.1038/s41467-023-41220-x)
Supplement: Supplementary file 1 — Supplementary Information [file 41467_2023_41220_MOESM1_ESM.pdf]

Supplementary Information

Evolutionary origin of genomic structural variations in domestic yaks

Liu X. *et al.*

This file includes Supplementary Figs. 1 to 12

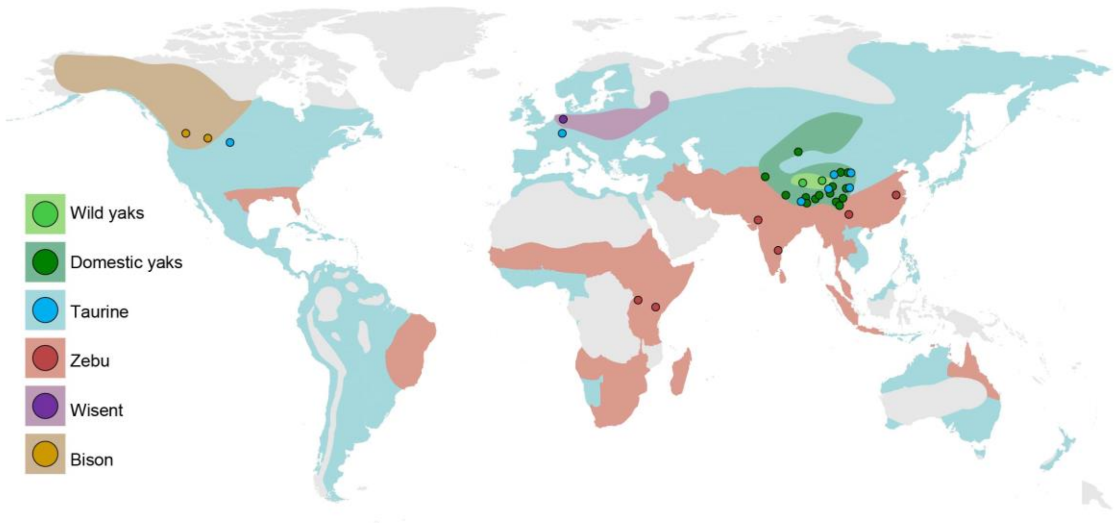

Supplementary Fig. 1 | Geographic distribution of the used bovine de novo genomes for phylogenetic analyses and construction of super-pangenome and graph-genome. The black circles filled with different colors represent the sampling locations of the *de novo* genomes. The world distributions of wild yaks, domestic yaks, taurine, zebu, wisent and bison are indicated by colors. Only 40 of the 47 genomic sampling positions from six bovine genera could be located to a precise geographical position.

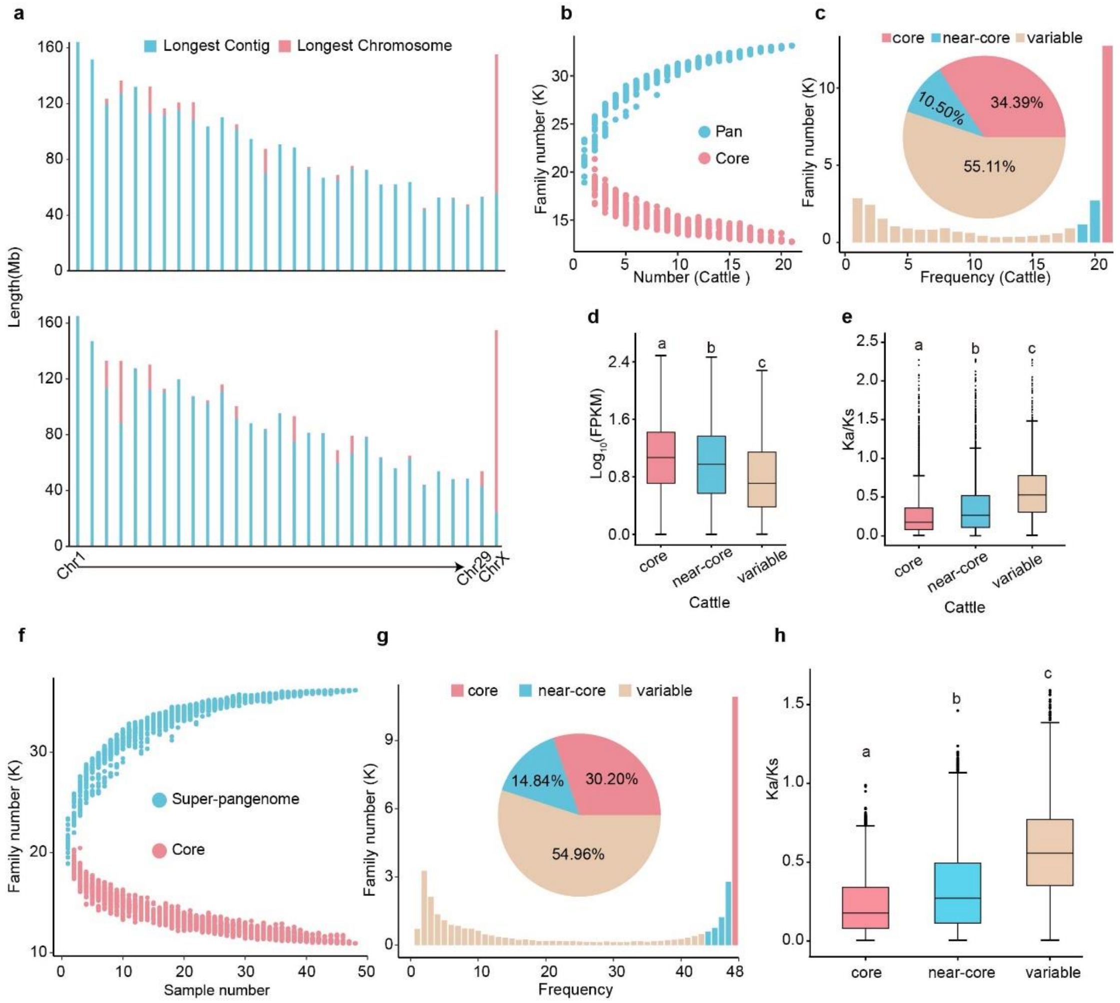

Supplementary Fig. 2 | Pan and core genome analyses of 21 cattle and 47 bovine genomes. a The total length of the longest chromosome (red) and the longest contigs (azure) in the pangenome assemblies representing 21 yaks and 7 cattle, respectively. **b, f** Counts of pan-gene and core-gene families with increased samples for cattle and 7 bovine species, respectively. **c, g** Numbers and frequencies of core, near-core, and variable genes for cattle and 7 bovine species, respectively. **d** The expression of core (13,251), near-core (7,132), and variable (3,751) genes for cattle. **e, h** *Ka/Ks* values of core (11,813; 10,755), near-core (5,017; 5,414), and variable genes (964; 1,305) for cattle and 7 bovine species, respectively. Lowercase letters indicate significant differences ($p<0.05$); two-sided Student's t-test. Box edges indicate the upper and lower quartiles, the centerlines indicate the median value, the horizontal bars indicate the maximum and minimum values, and the dots indicate outliers.

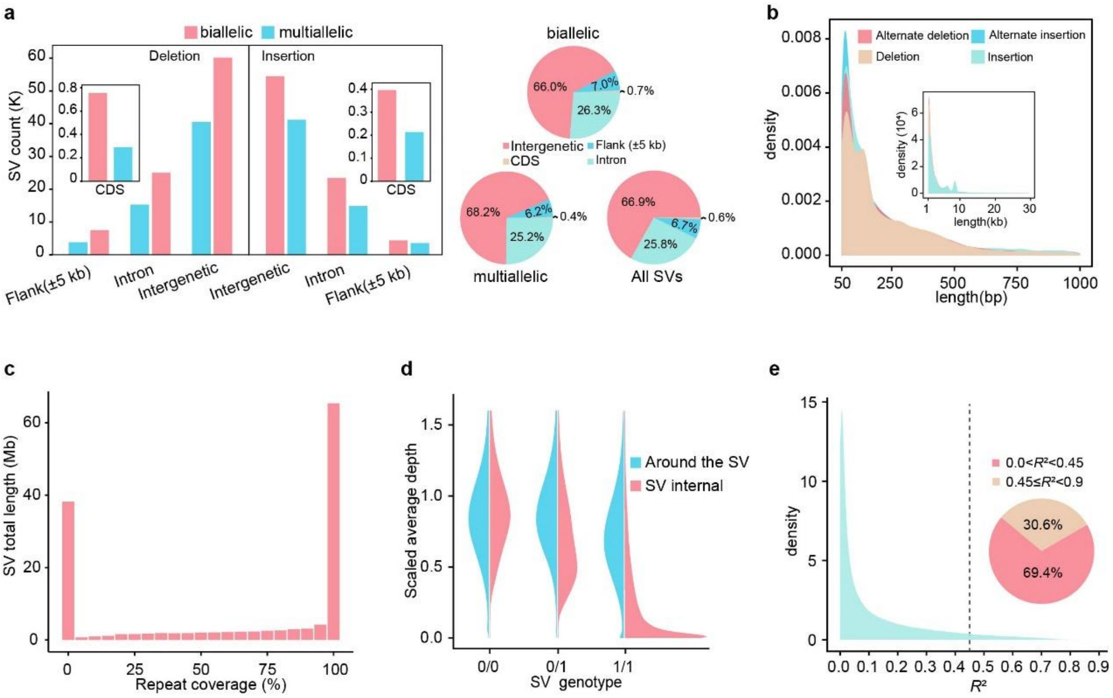

Supplementary Fig. 3 | Multi-assembly graph analyses of 47 bovine genomes. a. The bar and pie charts represent the distribution and percentage of the biallelic and the multiallelic SVs in different regions of the genome, respectively. **b** The length distribution of biallelic and the multiallelic SVs. **c** Distribution patterns of SV in genomic repetitive and non-repetitive regions. **d** Scaled average depth of SV-graph genotype based on population resequencing data. Haplotype 0 stands for the reference sequence, while haplotype 1 represents deletion. **e** The distribution of LD (R^2) between SVs and SNPs. For each SV, the R^2 with adjacent SNPs within 50 kb on either side is recorded. The grey dashed line indicates the threshold line (R^2=0.45, half of the maximum value).

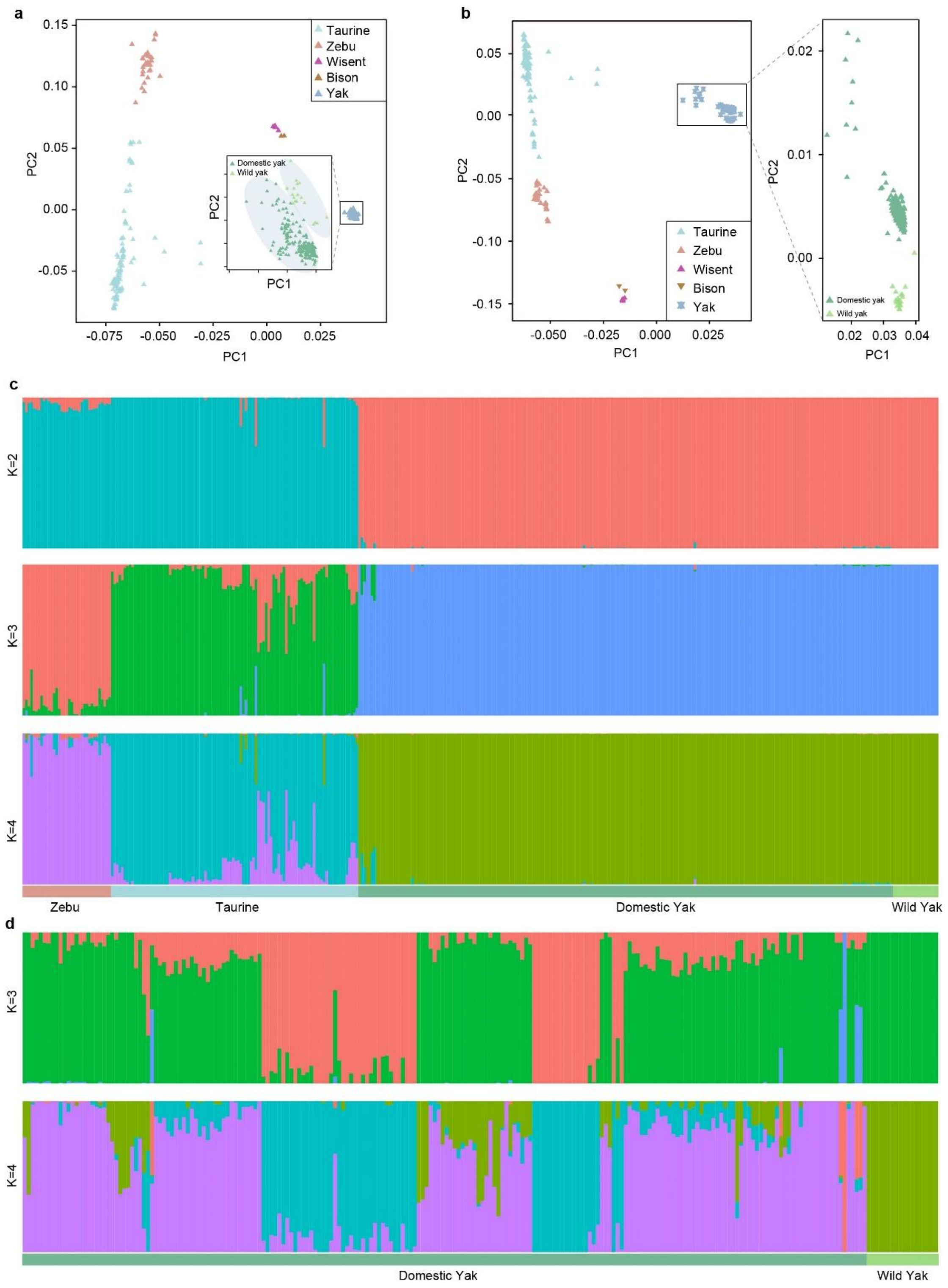

Supplementary Fig. 4 | Population genetic structure. Principal component analysis (PCA) of the distribution of all genotyped SVs (**a**) and SNPs (**b**) from all resquenced samples. (**c-d**) Population genetic structure of domestic yak, wild yak, taurine and zebu inferred from the admixture analyses using whole-genome SVs.

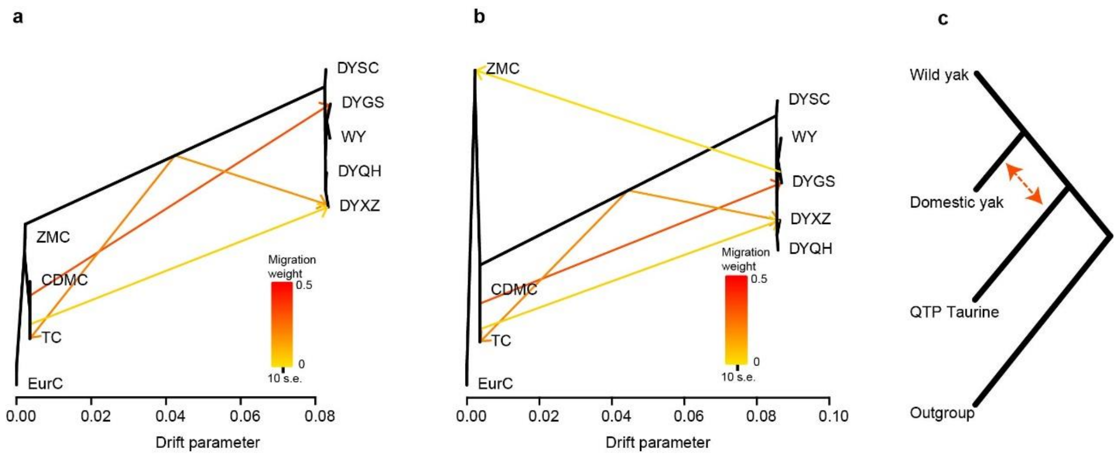

Supplementary Fig. 5 | Introgression analysis between domestic yaks and QTP cattle. **a** and **b** from TreeMix analysis and (**c**) confirmed by D-statistic analysis based on population whole-genome SVs. WY: wild yak, DY: domestic yak, EurC: European cattle, QTP cattle: TC (Tibetan cattle), CDMC (Chaidamu cattle), and ZMC (Zhangmu cattle).

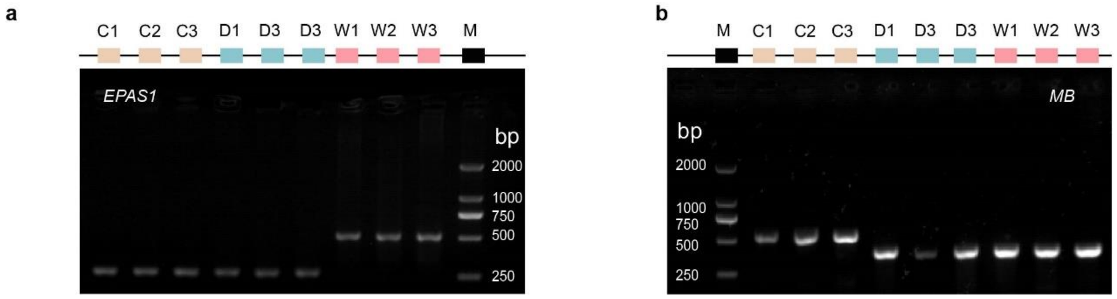

Supplementary Fig. 6 | Validation of the SVs. The electrophoresis diagrams of PCR products for two SV-haplotypes of *EPAS1*(**a**) and *MB* (**b**) in three domestic yaks, three wild yaks and three cattle. C: Cattle, D: Domestic yaks, W: Wild yaks, M: Marker.

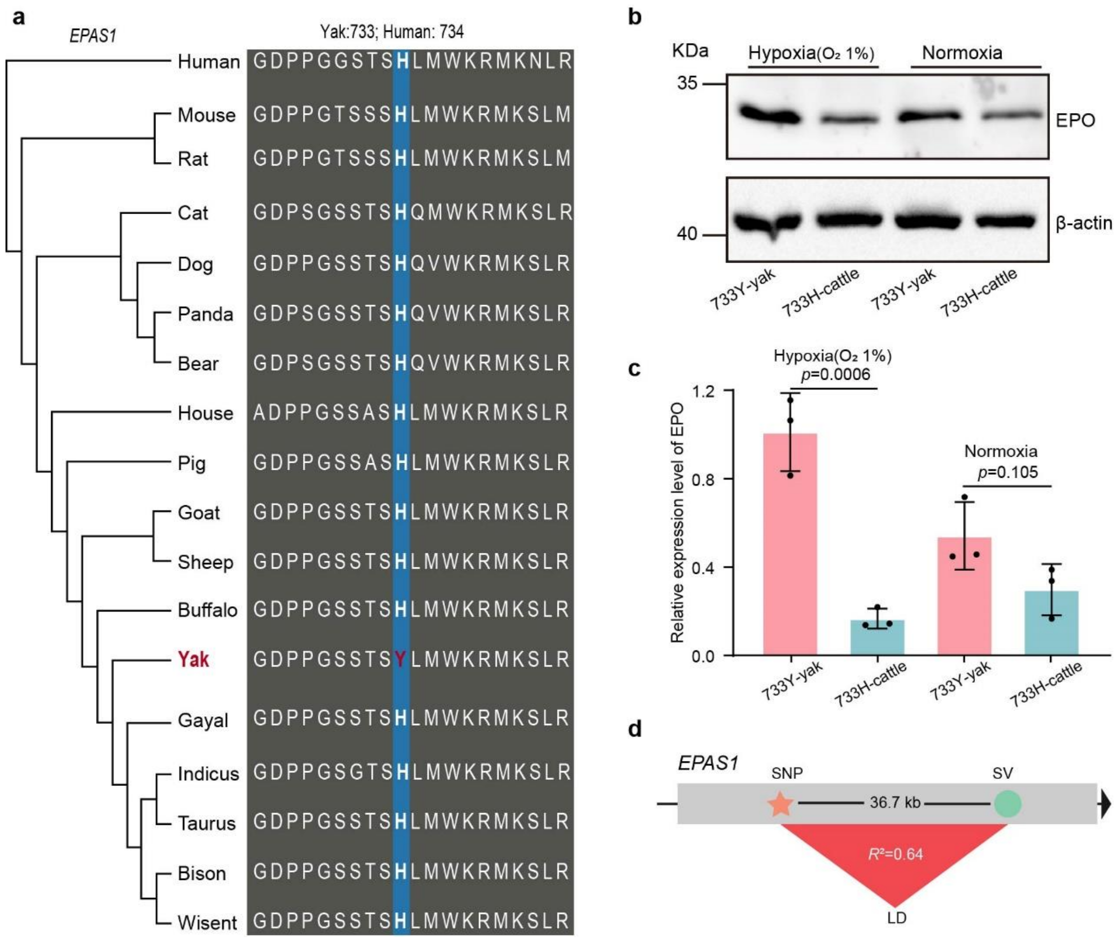

Supplementary Fig. 7 | Impacts of SNP on *EPAS1* has shaped environmental adaptation. a Variation in the EPAS1 protein among 17 species. The amino acid replacements in yak compared with all lowland species are shown in sky blue bar. **b** Western-blotting analysis of the *EPAS1* downstream gene *EPO* in 293T cells under hypoxia and normoxia. β-actin indicates the internal reference gene. See source data for original scans. **c** The mRNA expression levels of the *EPAS1* downstream gene *EPO* in 293T cells under hypoxia and normoxia detected by qPCR. Three biological duplications were conducted. Data are shown as mean ± SD. The *p*-value was calculated using Student's t-test (two-tailed). **d** The LD between SV and SNP on *EPAS1*. Source data are provided as a Source Data file.

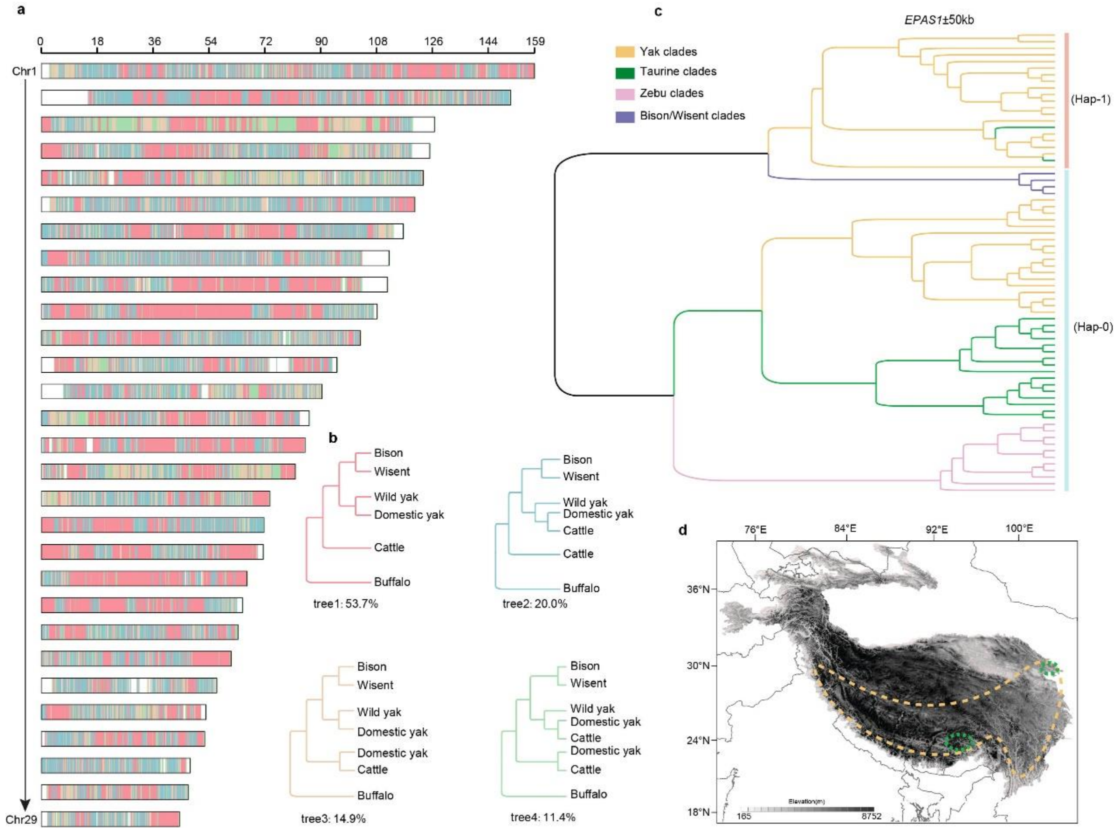

Supplementary Fig. 8 | Widespread interspecific SV introgressions between domestic yaks and cattle in the QTP.

a The distributions of all detected introgressed SVs in domestic yaks from cattle on the total genome. These introgressions were determined based on tree topologies established based on SNPs of the phased SV-nearby ± 50 kb haplotypes. **b** Four tree topologies and genic locations of SVs: no introgression between domestic yaks and cattle (tree1), introgression from domestic yaks to cattle (tree2), introgression from cattle to domestic yaks (tree3), and introgression between domestic yaks and cattle to each other (tree4). **c** introgression between domestic yaks and cattle with the tree based on SNPs of the 50 kb flanking regions of the phased *EPAS1* SV haplotype of using water buffalo as outgroup showing genetic exchange between cattle and yak. Hap-0 indicates the cattle type and Hap-1 indicates the yak type. **d** Geographical distribution of SV-haplotypes of *EPAS1* with yellow dashed line suggested with introgression in domestic yaks from cattle while the green dashed line for cattle with introgression from domestic yaks.

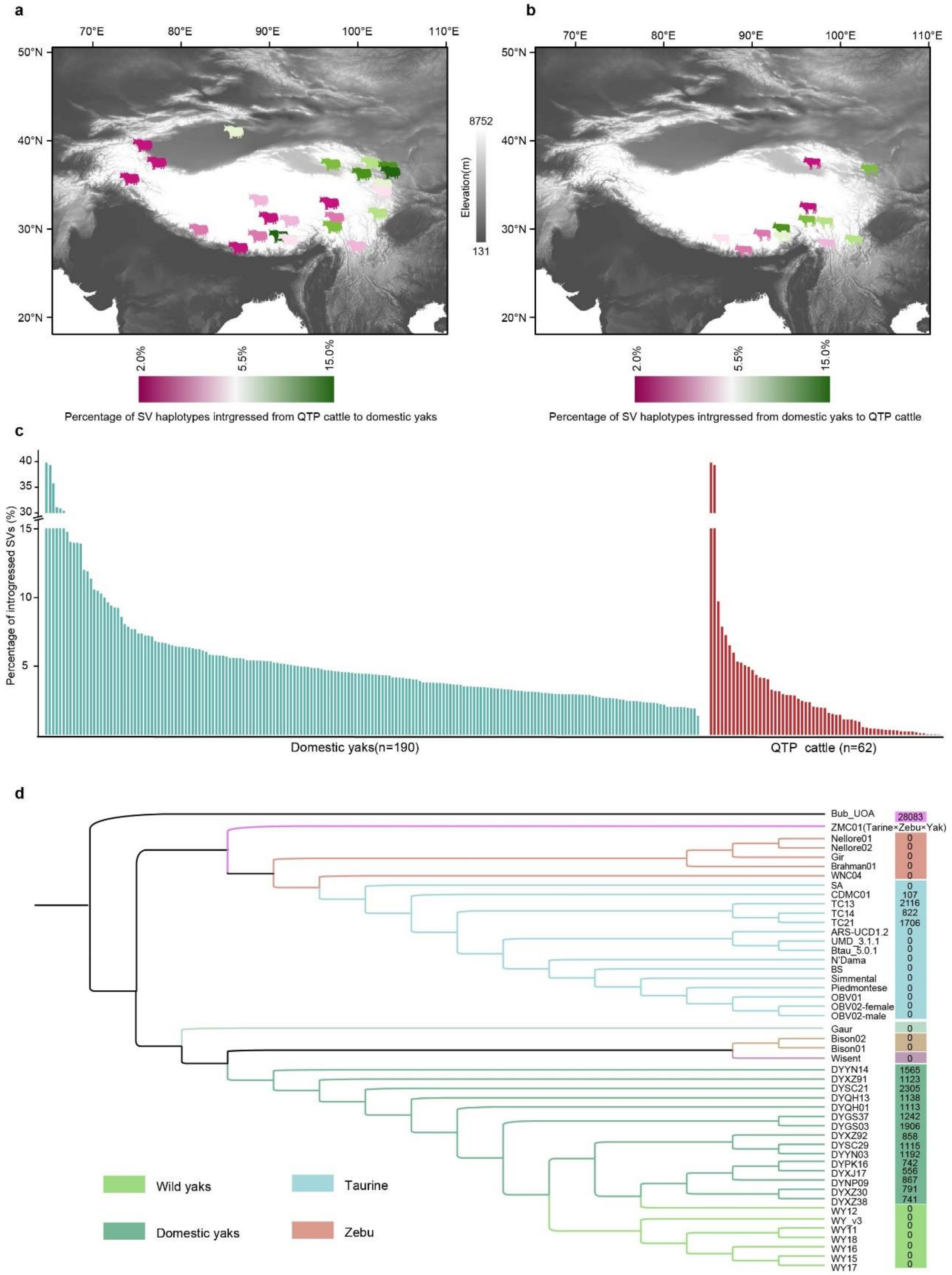

Supplementary Fig. 9 | The pattern distribution of SVs in which introgression occurred in individuals of the yak and QTP cattle.

a, b Domestic yaks and cattle in the QTP region have extensive introgression with each other. Averaging was used for multiple individuals in the same region. **c** The percentage of SVs in which introgression occurred in domestic yaks and QTP cattle. **d** The number of SVs introgressed in the *de novo* assemblies. The numbers in the bar graph represent the number of SVs that are introgressed.

CDMC: Chaidamu cattle, TC: Tibetan cattle, ZMC: Zhangmu cattle, WNC: Wannan cattle, BS: Brown Swiss, OBV: Original Braunvieh, SA: Sanga Ankole, Hereford cattle: ARS-UCD1.2, Btau_5.0.1 and UMD_3.1.1.

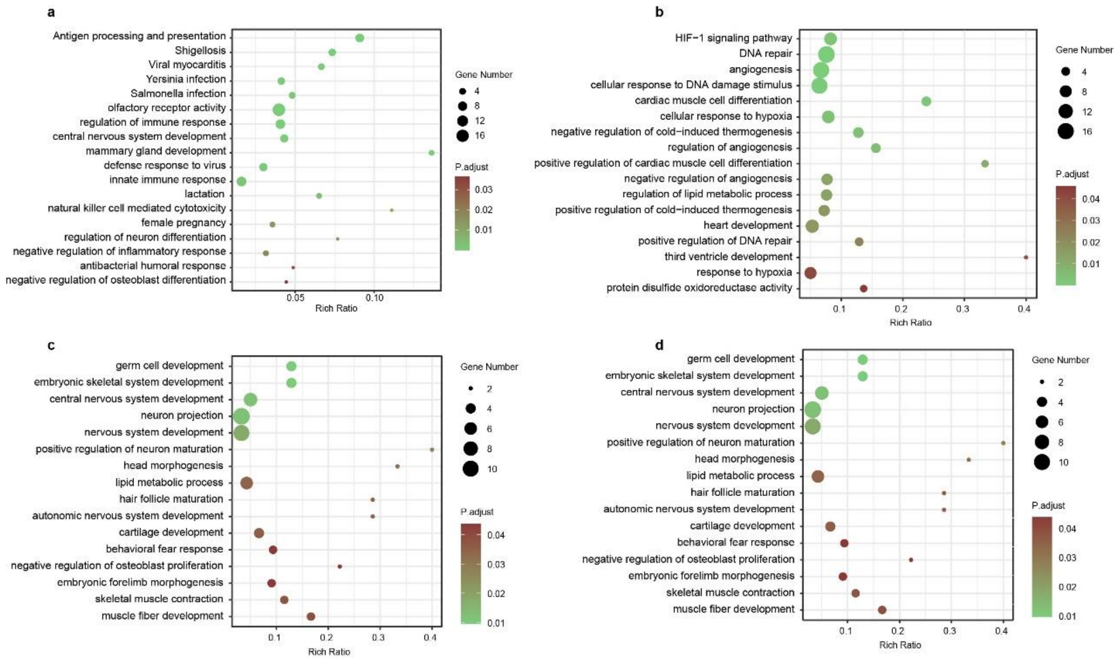

Supplementary Fig. 10 | a Functional enrichment analysis for new introgression genes from cattle to domestic yaks. **b-d** Functional enrichment analysis for introgression only from domestic yaks to cattle, only from cattle to domestic yaks and bidirectional introgression between domestic yaks and cattle. The hypergeometric distribution test was used for statistical testing. Significant enrichment was measured using corrected p-values.

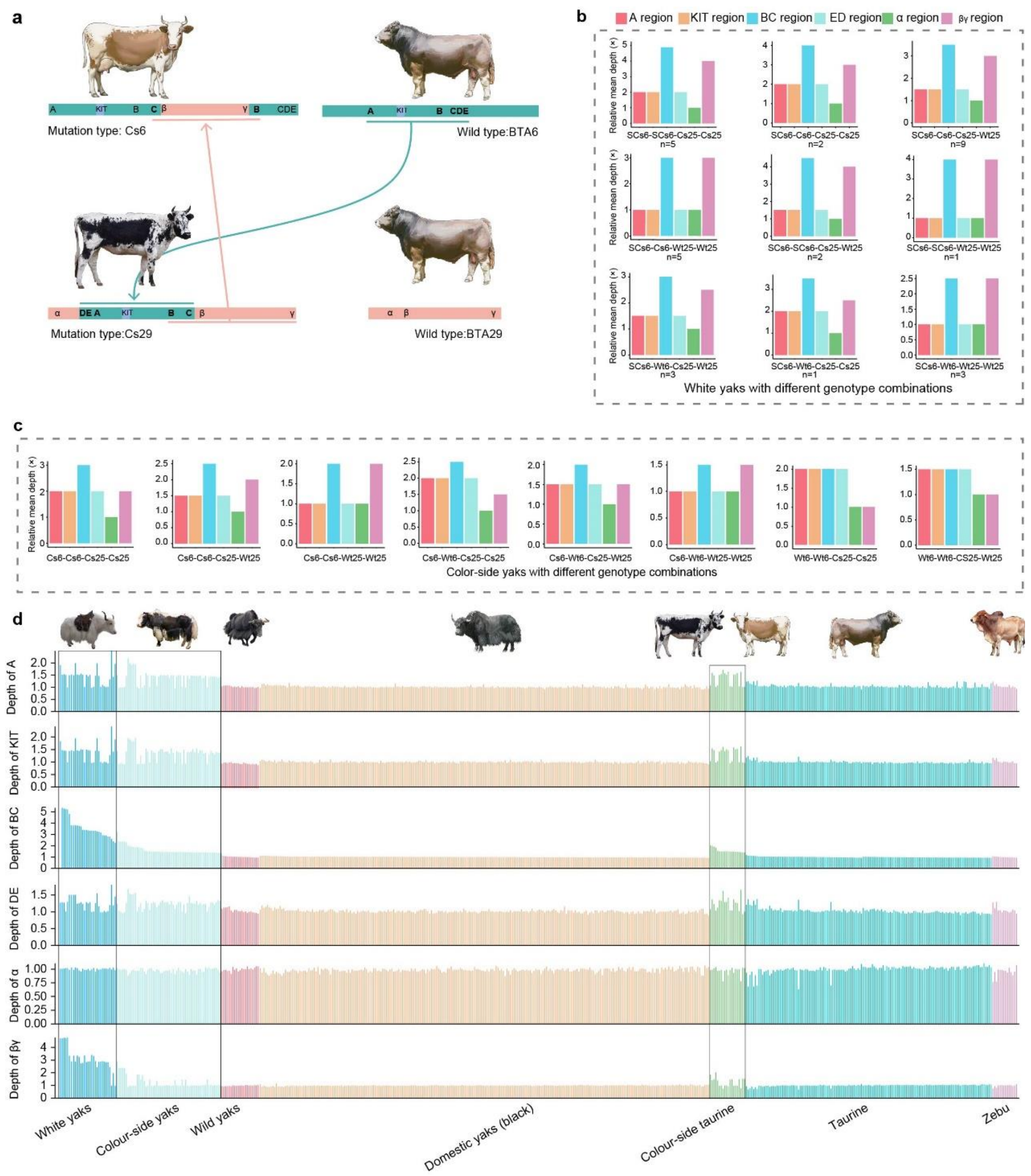

Supplementary Fig. 11 | Relative depth statistics of the mapping of yaks and cattle.

a Model for the generation of the cor-sided Cs29 and Cs6 of cattle alleles by serial translocation (Durkin et al., 2012). Relative depth displays of resequencing reads of different genotype combinations in white yaks **(b)** and color-sided yaks **(c)**. **d** Relative depth display of resequencing reads of different gene segment in yak and cattle individuals.

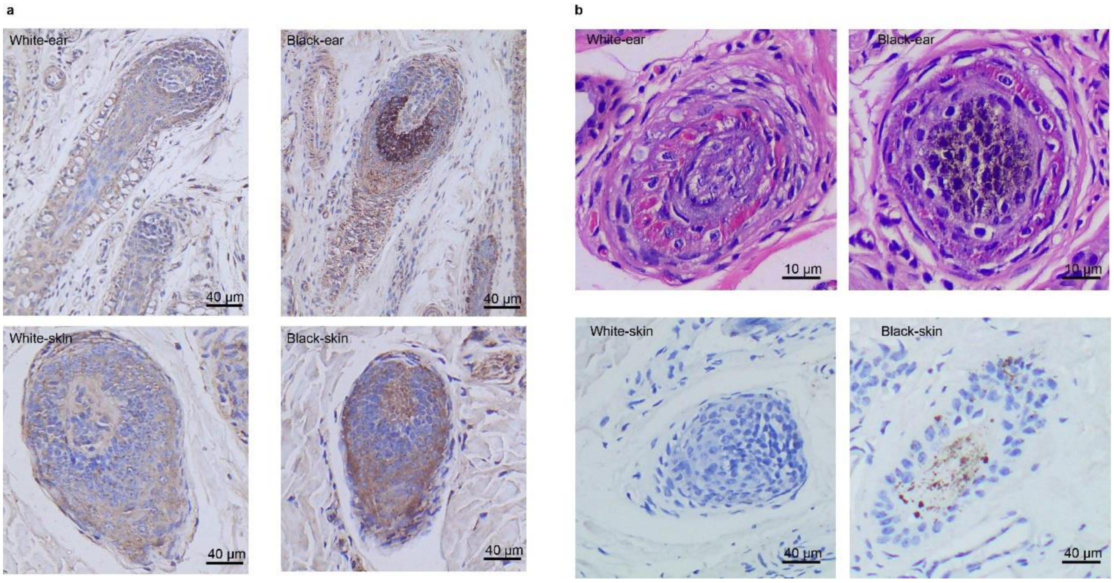

Supplementary Fig. 12 | Expression pattern of *KIT* in skin and ear tissues of white and black yaks. **a** Micrographs of immunohistochemistry of the KIT protein in sections of ear tissue (top) and croup skin (bottom) samples of white (left) and black (right) yaks. **b** Hematoxylin and eosin (Top) and DAB Detection Kit (bottom) staining of ear tissue (top) and croup skin (bottom) of black (right) and white (left) yaks. Images are representative of three experiments.
