# Supplementary material for: Evolutionary origin of genomic structural variations in domestic yaks
Source: Nat Commun. 2023 Sep 19;14:5617. doi: 10.1038/s41467-023-41220-x (PMC10509194; doi:10.1038/s41467-023-41220-x)
Supplement: Supplementary file 4 — Description of Additional Supplementary Files [file 41467_2023_41220_MOESM4_ESM.docx]

**Description of Additional Supplementary Files**

Supplementary Data 1: Resequencing information of the 386 samples.

Supplementary Data 2. Statistics of genomic sampling information of 47 bovine species.

Supplementary Data 3. Summary of sequencing data for 28 samples.

Supplementary Data 4. Quality assessments of 28 de novo assemblies.

Supplementary Data 5. The annotation estimation of 47 genome.

Supplementary Data 6. Statistics of node from graph pan-genome.

Supplementary Data 7. Different types of structural variations discovered from the multi-assembly graph.

Supplementary Data 8. Statistics on the enrichment of high-altitude adaptation gene for wild yaks.

Supplementary Data 9. SNPs with *F*_ST_ =1 with their overlapping genes between the wild yak and low-altitude bovine. Bolding indicates candidate pathways

Supplementary Data 10. Statistics of introgression of SV between domestic yaks and cattle in the gene regulatory region.

Supplementary Data 11. Functional enrichment analysis of introgression only from domestic yaks to cattle.

Supplementary Data 12. Functional enrichment analysis of introgression only from cattle to domestic yaks.

Supplementary Data 13. Functional enrichment analysis of bidirectional introgression between domestic yaks and cattle.

Supplementary Data 14. Functional enrichment analysis of genes associated with domestication-related SVs.

Supplementary Data 15. SNPs for *F*_ST_ abnormalities (> 0.3) between wild and domestic yaks and their overlapping genes. Bolding indicates candidate pathways

Supplementary Data 16. List of 178 RNA-seq data for yaks and cattle.
